# Early Warning of Infectious Diseases in Hospitals Based on Multi-Self-Regression Deep Neural Network

**DOI:** 10.1155/2022/8990907

**Published:** 2022-08-18

**Authors:** Mengying Wang, Cuixia Lee, Wei Wang, Yingyun Yang, Cheng Yang

**Affiliations:** ^1^State Key Laboratory of Media Convergence and Communication, Communication University of China, Beijing, China; ^2^Peking University Third Hospital, Beijing, China; ^3^Goodwill Hessian Health Technology Co, Ltd., Beijing, China

## Abstract

**Objective:**

Infectious diseases usually spread rapidly. This study aims to develop a model that can provide fine-grained early warnings of infectious diseases using real hospital data combined with disease transmission characteristics, weather, and other multi-source data.

**Methods:**

Based on daily data reported for infectious diseases collected from several large general hospitals in China between 2012 and 2020, seven common infectious diseases in medical institutions were screened and a multi self-regression deep (MSRD) neural network was constructed. Using a recurrent neural network as the basic structure, the model can effectively model the epidemiological trend of infectious diseases by considering the current influencing conditions while taking into account the historical development characteristics in time-series data. The fitting and prediction accuracy of the model were evaluated using mean absolute error (MAE) and root mean squared error.

**Results:**

The proposed approach is significantly better than the existing infectious disease dynamics model, susceptible-exposed-infected-removed (SEIR), as it addresses the concerns of difficult-to-obtain quantitative data such as latent population, overfitting of long time series, and considering only a single series of the number of sick people without considering the epidemiological characteristics of infectious diseases. We also compare certain machine learning methods in this study. Experimental results demonstrate that the proposed approach achieves an MAE of 0.6928 and 1.3782 for hand, foot, and mouth disease and influenza, respectively.

**Conclusion:**

The MRSD-based infectious disease prediction model proposed in this paper can provide daily and instantaneous updates and accurate predictions for epidemic trends.

## 1. Introduction

Infectious diseases are usually characterized by rapid transmission, high morbidity, and high uncertainty and are extremely dangerous. The global health sector is currently working to promote early warning and surveillance capabilities for infectious disease outbreaks. Medical institutions are the frontline units for detecting, reporting, and treating patients with infectious diseases, and they are also responsible for the routine diagnosis and treatment of noncommunicable diseases to ensure a harmonious society [1]. China has established a relatively well-developed national infectious diseases information monitoring system (NIDIMS), owing to which the earlier practice of cascading reporting of infectious diseases has been changed to direct reporting to the government [1].

Through the NIDIMS, infectious diseases are reported directly to the government [1, 2]. A total of 40 types of infectious diseases are covered, including two types of class A infectious diseases, 27 types of class B infectious diseases, and 11 types of class C infectious diseases [1]. As early as the end of 2006, the direct reporting network covered 100% of disease control centers, 95% of medical and health institutions at the county level and above, and 70% of rural health centers nationwide [2], making the reporting of infectious diseases 10 times faster [3]. In 2020, COVID-19 exposed the disadvantages of the system: it was not equipped with active alerting and intelligent analysis of predetermined warnings [4], which impedes the timeliness of diagnosis and the accuracy of risk prevention and control, resulting in the possibility of underreporting or delayed reporting [3]. The existing infectious disease warnings are also mostly government warnings regarding the national situation. It is difficult to provide actual feedback on the prevailing situation in local medical institutions as it develops, and there is a lack of efficient early warning based on the data obtained from medical institutions. As a result, the existing system cannot support the deployment of hospitals for epidemic prevention and control, which will substantially weaken the role of general hospitals as frontlines for epidemic prevention and control.

## 2. Related Work

To address these issues, most of the existing studies adopt polynomial fitting, mathematical statistical models, SEIR methods, and machine learning. Dette et al. [5] applied a polynomial function to fit the curve, which is a traditional statistical method that uses only the data series of the number of confirmations. By increasing the number of polynomials, it is possible to fit a more complex function curve that cannot be predicted flexibly in practice according to the current time. At present, infectious disease dynamics models are also widely applied, such as SEIR model, susceptible-exposed-infected-recovered-die-hard-infected (SEIRD) model, and their variants. For example, Ghostine et al. [6], Wangping et al. [7], Wei et al. [8], Yang et al. [9], and Youssef et al. [10] adopted the SEIR method to predict the spread of COVID-19. Despite the small number of parameters in the kinetic model, the actual meaning of the parameters is explicit, and the level of interpretability is high. However, there must be multiple types of data available, including the latent population, the number of recovered patients, and other group data that are difficult to obtain. For example, Feng et al. [11] obtained the latent data by estimation, which affects accuracy, thus restricting the scope of application. In some studies, the autoregressive integrated moving average (ARIMA) model [12, 13], linear regression [14], moment estimation [15], hidden Markov model [16], and grey self-memory coupling model [17] were adopted. However, there is a limit to the scope of application for each of these mathematical models, which means they are often suited to a single or certain type of disease. Not only does the data used in the research have a single dimension, but it is also heavily reliant on the information obtained from epidemiological retrospective surveys. Erraguntla et al. [18] and Telarolli et al. [19] treated the data series of developmental trends exhibited by infectious diseases as random ones, and the autoregressive model was adopted to analyze the interdependence and autocorrelation between various random variables. However, stable time series are required for this method. In the modeling process, only the number of deaths and that of people who have recovered are used, while the information other than the numerical sequence is ignored.

Since the outbreak of the COVID-19 pandemic, there are many scholars [20–24] from various countries who have studied machine learning and neural network to predict the developmental trend of COVID-19 and other infectious diseases. However, this method is disadvantaged by long-time series and the lack of data regarding important influencing factors in the developmental trend of infectious diseases, such as environment and climate. As a result, the prediction results can only be obtained on a monthly basis and are prone to overfitting. Gu et al. [25] used three-layer long short-term memory (LSTM) to model the developmental trend of hand, foot, and mouth disease, taking into account various external factors such as wind speed and temperature, with the root-mean-square error (RMSE) reaching 0.71. However, for children with obvious group characteristics, factors such as opening and closure of schools is not considered. Liao et al. [26] applied a neural network to learn the parameters in the dynamic model, which accelerates the adjustment of the parameters used in the dynamic model. However, in the process of parameter learning, only the product coefficient can be learned when there are multiple coefficients used in the dynamic model. Therefore, it is difficult to decompose multiple coefficients, which affects the interpretability. Bedi et al. [27] used the SEIR dynamic model and LSTM to study COVID-19, with satisfactory prediction results. However, constraints such as difficult access to population and limited data dimensions are yet to be addressed.

Considering the advantages and disadvantages of existing methods, an MSRD-based approach is proposed in this study to predict the developmental trend of infectious diseases. While incorporating the information on multi-dimensional epidemiological features closely associated with infectious diseases, the proposed model simultaneously adopts LSTM as the building block to construct a recurrent neural network. In addition, temporal modeling and calculation are performed for the time-series data organized by means of self-regression learning, which addresses the challenges facing existing studies, such as the difficulty in obtaining quantitative data such as latent population, the overfitting of long time series, and the lack of consideration given to the epidemiological features of infectious diseases and only a single series of the number of patients. In addition, compared with the SEIR model, machine learning model, and neural network model in existing studies, the MSRD-based approach proposed in this study achieves better performance.

## 3. Methods

### 3.1. Sources of Data

The data comes from two sources. One is the official monthly public health scientific data of the national center for disease control and prevention (CDC) from 2012 to 2017. The second is the daily data of inpatient and outpatient medical records from Peking University Third Hospital from 2012 to 2020. The hospital data was obtained from the hospital data center. The hospital data center adopts Hadoop architecture and integrates a Hadoop distributed file system, HBase column database, and Hive data warehouse, which can easily perform data storage and analytical computations [28]. After in-depth mining of 110,000 historical data points on infectious diseases accumulated in a large hospital over eight years, big data technology is applied to collect and clean the clinical data and then store and manage them in a centralized way, which provides the necessary basis for the training and application of an early-warning model for infectious diseases. In addition to the data from the data center, this study collected the daily temperature, humidity, wind, and other climate data from the website of the national meteorological data department, given the close relationship between some infectious diseases and climate factors [29, 30]. In addition to the aforementioned number of infectious diseases and environmental factors, the spread of infectious diseases is closely related to human activities. For example, infectious diseases are more likely to spread widely in human aggregated activities. Therefore, this factor is also used as a data feature for prediction in this study, which is reflected in the study of some infectious diseases.

The study was approved by the Medical Science Research Ethics Committee of Peking University Third Hospital (serial number: IRB00006761-M2020318). All methods were performed in accordance with the relevant guidelines and regulations.

### 3.2. MSRD Model

**Figure 1:**
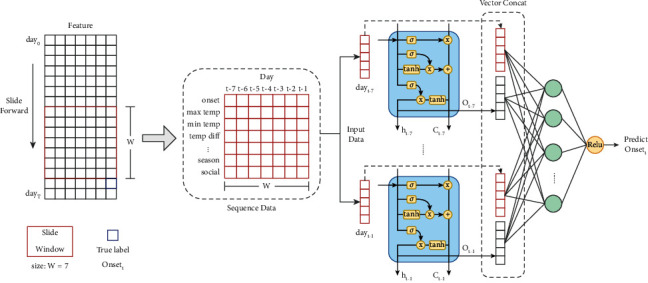
Multi self regression deep model.

In this study, we constructed an MSRD model using an LSTM neural network and a sliding window as the core structure, as shown in Figure 1. The MSRD model takes the time-series data of multiple elements from multiple days as inputs to predict the number of confirmed infectious diseases on the next day. The trend of this number confirmed that infectious diseases can be generated through continuous prediction. LSTM consists of an input gate, a forgetting gate, and an output gate, which can be used to preserve information and calculate the features of data with time-series characteristics. This study uses daily data as the experimental metadata. Each metadata is an *m* × 1 vector that includes the number of confirmed infectious diseases on a particular day, the current date, current climate data, and the expected social activities. The data in the experiment includes D-Day. The total data occupies an m*∗D* matrix. MSRD uses a window of size m*∗w* to slide to the right within the total data of m*∗D*. Each slide extracts fragment data of size m*∗w* as the input data and takes the confirmed number of infectious diseases in the first column of the *m* × 1 vector outside the window as the label data. At the end of sliding, *D* − *w* pieces of m*∗w* input data matrix and *D* − *w* label data can be obtained, as shown in the sliding window in Figure 1. Then, the *D* − *w* pieces of m*∗w* data are fed into the LSTM neural network, and the LSTM can learn the mapping function between the m*∗w* segment data and the corresponding label. Next, to make use of the output of the LSTM neural network and the characteristic information of the original data simultaneously, the input data of m*∗w* is expanded in a one-dimensional form and horizontally spliced with the output of the LSTM neural network, followed by the prediction result of the diagnosis number being output through the feedforward neural network with a rectified linear unit (ReLU) function as the activation function. Based on the original fragment data containing multi-day data, the MSRD model uses the unique structural characteristics of LSTM to extract the time-series features in the data. It can simultaneously use the historical time-series data and the multi-dimensional feature data outside the series to predict the epidemic trend of infectious diseases. Compared with the typical LSTM that uses only the output of the last time step, MSRD makes full use of the output of each time step of the sequence structure based on the use of time windows to extract data and feeds the original first-order data (not computed by LSTM neurons) and second-order data (computed by LSTM) together into a feedforward neural network with multiple hidden layers, enhancing the crossover capability of the features as well as the model fitting ability. In addition, the introduction of the time window concept improves the flexibility of the model in predicting infectious diseases and avoids the overfitting problem caused by using long series to train the model, allowing the model to make more accurate predictions for the future using data from different date spans according to different application scenarios.

### 3.3. Evaluation Metrics

We evaluate the performance of the proposed MSRD method and compared it with the three other models using the MAE, which is the simplest measure of fitting and prediction accuracy describing the mean value of the difference between the model prediction results and the true results at each time in terms of the series as a whole. In addition, we also use the RMSE to measure the deviation of the observed values from the ground truth, which is calculated as follows:

(1) $$\begin{array}{c} \text{MAE}=\frac{1}{n}\sum_{t=1}^{n}\left|\text{actual}\left(t\right)-\text{predict}\left(t\right)\right|, \end{array}$$

(2) $$\begin{array}{c} \text{RMSE}=\sqrt{\frac{1}{n}\sum_{t=1}^{n}{\left[\text{actual}\left(t\right)-\text{predict}\left(t\right)\right]}^{2}}. \end{array}$$

### 3.4. Statistical Software

We used Microsoft Excel 2016 to construct the original database and used *Python* v3.6.4, PyTorch v1.6.0, Numpy v1.14.1, and Scikit-Learn v0.19.1 for model building.

## 4. Experimental Results

### 4.1. Trends in the Incidence of Real Infectious Diseases in Hospitals

**Figure 2:**
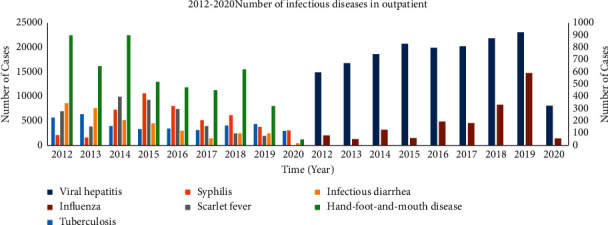
Number of infectious diseases in outpatients between 2012 and 2020.

**Figure 3:**
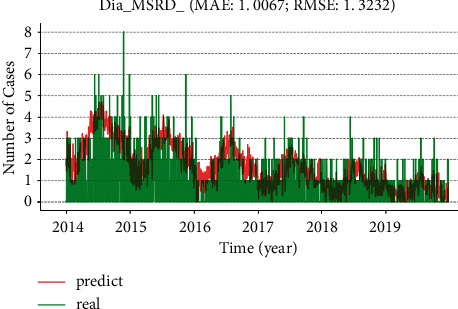
The MSRD model predicts the epidemic situation of infectious diarrhea in 2021. Overlapping contrast: the green line denotes the real data, and the red line shows the predicted data.

Based on real hospital outpatient data collected from the Peking University Third Hospital from 2012 to 2020, we analyze seven common infectious diseases, including hand-foot-and-mouth disease (HFMD), influenza, viral hepatitis, infectious diarrheal disease, scarlet fever, syphilis, and tuberculosis during in this study, as shown in Figure 2. Among them, influenza and viral hepatitis have a trend of slow growth year by year, while HFMD, infectious diarrheal disease, scarlet fever, syphilis, and tuberculosis have decreased year by year, which sufficiently demonstrates the effectiveness of overall infectious disease prevention and control, but there is a trend of slow growth for influenza and viral hepatitis, and the government should take preventive measures in advance. The year 2020 is more affected by the COVID-19 epidemic, and the actual number of hospital visits has also decreased significantly. Based on the analysis of data between 2012 and 2020, we construct an MSRD model, where we tested multiple parameter combinations: the sliding window length *w* candidates (3, 5, 7, 9, 14), the number of LSTM neurons candidates (6, 8, 16, 32, 64, 128), the number of feedforward neural network neurons candidates (32, 64, 128, 256, 512), and multiple learning rates. The optimal values of the above parameters are as follows: the sliding window length is 7, the number of LSTM neurons is 16, the feedforward neural network neuron is 128, and the learning rate is 0.001. It was observed that as the sliding window length, the number of LSTM neurons and the number of feedforward neurons increased and the performance of the MSRD model in the training data improved, but the performance in the test set first increased and then decreased. The results demonstrate that the model complexity and the number of days of historical data used are positively correlated with the fitting ability of the model, while the generalization ability varies, into a trend of first increasing and then decreasing.  Figure 3 shows the model trained using the optimal parameters described above to predict the prevalence of infectious diarrhea in 2021. The model predicts a continuous decrease in the number of infectious diarrheas in 2021. See Supplementary Material 1 for the prediction results of other infectious diseases.

### 4.2. Comparison of MSRD and SEIR Models

**Figure 4:**
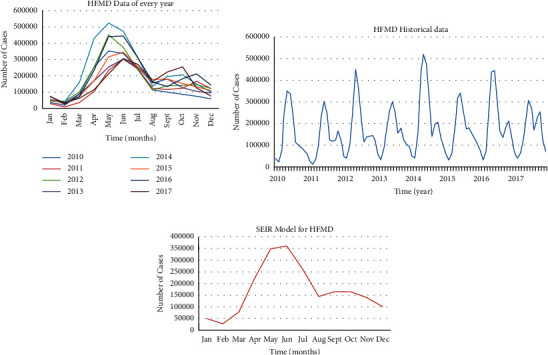
National monthly real data curve (a), continuous curve of monthly real data over the years in China (b), and SEIR model fitting curve (c).

In the research of infectious disease prediction, SEIR kinetic model is quite common. The main idea is to divide the whole population into different groups in a closed system and design the population transfer coefficients among different groups according to the infectious disease transmission mechanism, so as to form the differential equations. According to the National Data Center for Public Health Sciences, we build the SEIR model for HFMD. As shown in Figure 4, the model has monthly granularity. We observe that the number of HFMD cases in the national data shows a consistently increasing trend every year. The prediction trend after model learning is identical to the historical development trend. Therefore, it is feasible to use national data modeling. However, the national data alone has no daily data per month, while the fine-grained data are insufficient.

**Figure 5:**
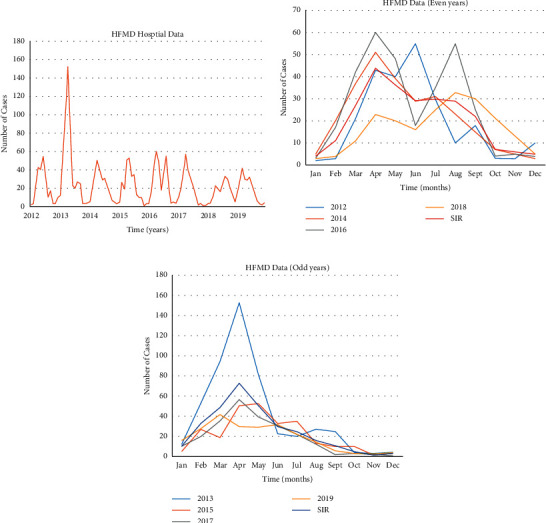
HFMD monthly curve of real data of the hospital (a), real and fitting data curve of even years and months (b), and real and fitting data curve of odd years and months (c).

**Figure 6:**
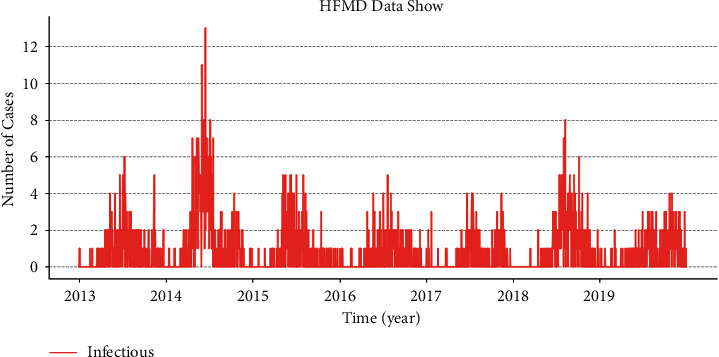
Daily data of real HFMD cases in the hospital.

Based on the data of a hospital, here, we take HFMD as an example and use the “monthly granularity” for modeling, as shown in Figure 5. It is difficult to obtain the data of the exposed persons, which is necessary for SEIR. The SEIR model is used to model by stages, the results of which are given below.  Figure 6 shows the daily data of real HFMD cases in the hospital.

It is clear that the SEIR model is poorly fitted to the infectious disease data of medical institutions for the following reasons: (1) there is an evident lack of continuity of infectious diseases in medical institutions. In the data of daily granularity, there are many months in which there are zero cases of HFMD. (2) The SEIR model requires population information such as susceptible groups, latent groups, isolated groups, infected groups, recovered groups, and dead groups, among which susceptible groups can be estimated; however, infected groups and recovered groups are very important, and these data are difficult to obtain in medical institutions. (3) For the infectious disease data of medical institutions with a small magnitude, the “daily granularity,” according to the single-digit change, leads to significant fluctuations in the data curve. Therefore, the SEIR model is suitable for trend forecasting with a large order of magnitude and comprehensive population information and is not applicable to forecasting on the basis of medical institutions.

### 4.3. Prediction Effect of MSRD

Based on the MRSD model and careful feature engineering, it is eventually applied to the training of the model. Feature engineering is the processing of data for the purpose of maximizing the extraction of effective features from the raw data for use in algorithms and models. For the current study, the number of daily confirmed cases of each infectious disease from January 1, 2012, to December 31, 2019, was first counted from the hospital data center. After that, the current year, month, and season are extracted from them to identify the current year, month, and season based on the date. The year and month are characterized by numerical type, and the season is represented in the form of a unique heat code. Next, the collected climate data are processed to calculate the diurnal temperature difference of the day. Finally, considering that infectious diseases are closely related to human group activities, another feature of social factors is incorporated into the training data. For example, HFMD is a prevalent infectious disease among children and students, and the main group activity of adolescents is studying at school, so the feature of whether they are currently on vacation is added to the features to characterize the social gathering activity.

The features for training include the daily number of confirmed cases of the target infectious disease, the current year, the current month, the current season, the highest temperature of the day, the lowest temperature of the day, the temperature difference of the day, whether the students are currently on vacation, and so on. The above features are then constructed as time-based series data. The features we selected are based on the literature review [6, 18, 19, 25, 29, 31–33] and expert knowledge. The highest and lowest temperatures within a single day in conjunction with the season can describe the climate. The development trend of certain infectious diseases is clearly known to be related to the environment and climate change. Consequently, the introduction of these features can represent the prevalent environmental climate to a certain extent and thus correlate with the development trend of infectious diseases. The feature “intraday temperature difference” is introduced based on the disease characteristics of infectious diseases. For example, when the intraday temperature difference is large, people are more vulnerable to influenza. The feature “students are on holiday” represents social activity factors, in order to account for the fact that socializing can lead to the spread of infectious diseases. The features used in this study combine the climatic environment, the characteristics of infectious diseases, and social activity factors. In addition, in light of epidemics of infectious diseases that occurred in the past, comprehensive consideration from multiple perspectives can better help improve the prediction effect of the prediction model.

**Figure 7:**
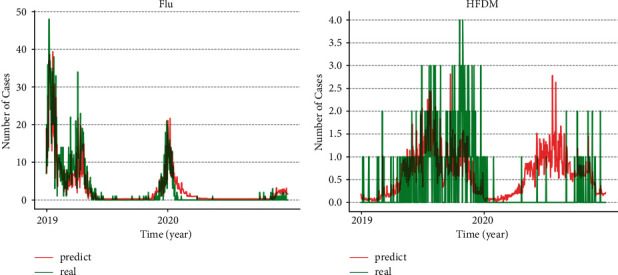
(a) Comparison of the real and predicted trends of influenza and (b) comparison of real and predicted trends in HFMD.

Finally, for the training of the MSRD model and the validation and evaluation of the model's effectiveness in predicting future infectious disease epidemic trends, data from October 28, 2013, to December 31, 2018, with a total of 1,890 time-series samples, accounting for 82.7% of the total time-series sample data, were used as training data in the study; a total of January 1, 2019, to January 31, 2020, 395 time-series samples were used as the test data.  Figure 7 shows the test results of applying the MSRD model to predict the epidemic trends of HFMD and influenza. The MSRD model is selected with a sliding window length of 7; the number of LSTM neurons is 32; the number of feedforward neural network neurons is 128; and the model is trained at a learning rate of 0.001. The predicted infectious disease epidemic trends from the multi-dimensional autoregressive neural network model shown in the figure broadly match the actual trends. The MAE was used to evaluate the results in the regression prediction. In this study, the number of confirmed cases of HFMD and influenza were of different orders of magnitude, and the MAE of the multi-dimensional autoregressive neural network was 0.6928 and 1.3782 cases lower for the test data in the training of both, which means that the average difference between the number of diseases predicted by the model at each time and the real number of diseases on a corresponding day were 0.6928 and 1.3782 cases, respectively. The difference between the prediction results of HFMD and the trend of influenza is mainly attributable to the magnitude of the total number of cases being different and a higher number of breakpoints in the real data of HFMD. The highest number of single-day influenza cases is nearly 50, while that of HFMD is only 10. At the same time, there is not only a substantial discrepancy between the data of HFMD and influenza but also a large number of zero cases in the time series of HFMD data. As a result, the continuity of data is poor, thus making it more difficult for the model to capture the regularity of data.

## 5. Discussion

### 5.1. Analysis of Model Methods

The MSRD model proposed in this paper provides a better prediction result than the SEIR model. The SEIR dynamic model is suitable for the prediction of large orders of magnitude and the entire population, and the influence coefficient in the model needs to be set manually, so it is difficult to tune the parameters. Consequently, it cannot be used to flexibly predict based on the actual conditions. In addition, SEIR struggles to fit infectious diseases without obvious regularity. During the prediction of the hospital's own data, the proposed MSRD method is set to “day,” and the features of multiple high correlation dimensions, including multiple infectious diseases, current environmental conditions, and transmission factors, are calculated with better nonlinear fitting ability. The MSRD model can flexibly predict real infectious diseases according to current conditions and factors at any time while obtaining better prediction results.

### 5.2. Model Performance Comparison

**Figure 8:**
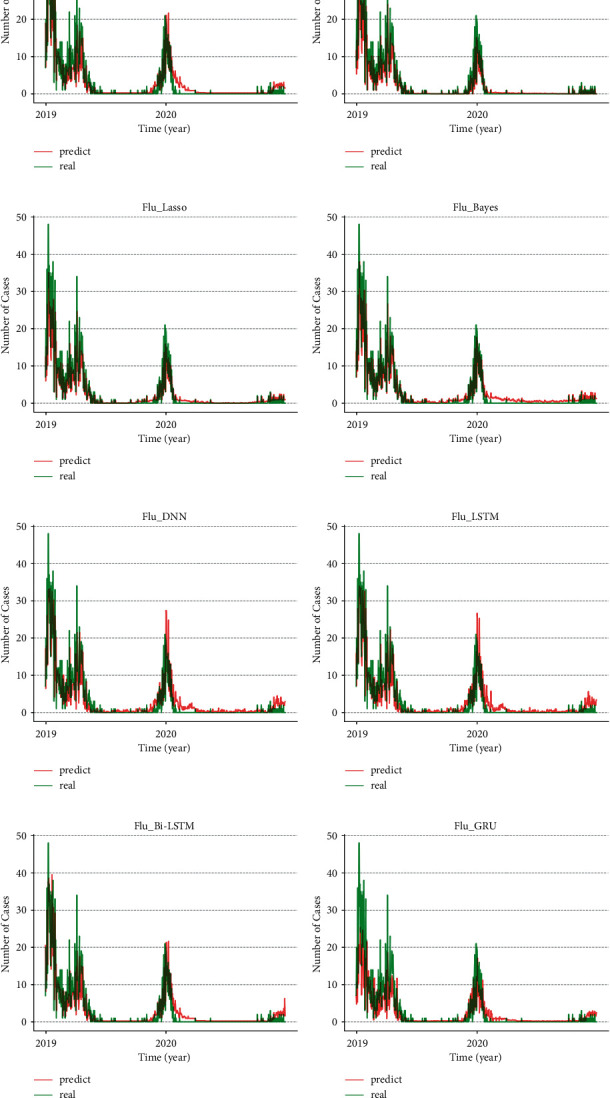
(a) MSRD model results, (b) SVM model results, (c) Lasso model results, (d) Bayesian model results, (e) DNN model results, (f) LSTM model results, (g) Bi-LSTM model results, and (h) GRU model results.

To verify the performance of the MSRD model, we compared the MSRD method with a variety of commonly used regression prediction models, such as support vector machine (SVM) [34], Lasso regression (lasso) [35], and Bayesian method (Bayesian) [36–38] aiming at the trend prediction of influenza and HFMD. Among them, the Bayesian regression method is less common, and this model assumes that the prior probability, likelihood function, and posterior probability are normally distributed. In this calculation, we need to maximize the marginal likelihood function to estimate the model parameters and regression coefficients. The above comparison models can be found in Python's scikit-learn library. In addition, the deep learning models that are used in the prediction of infectious disease trends are also compared: deep neural network (DNN) [11, 26], LSTM [20], bi-directional long short-term memory (Bi-LSTM), and gated recurrent unit (GRU). Among them, the MSRD model selects a sliding window length of 7, the number of LSTM neurons as 32, and the number of feedforward neural network neurons as 128. The model is trained at a learning rate of 0.001, with 223 epoch iterations being performed. The process of getting the best parameters of MSRD is provided in Supplementary Material 2. From Figure 8, it can be found that the performance of the proposed MSRD method in the two diseases is the best, making it an excellent model for practical applications. As can be seen from Table 1, the MSRD prediction results for different diseases had significant differences, and the data used in each model were identical. The reason is that the models have different learning and fitting ability to the data, and the essence is that each model has a different structure, complexity, and computational principle.

### 5.3. Impact of Infectious Disease Data Sources on Model Prediction

For hospitals, the probability of infectious diseases is lower than that of noninfectious diseases, and the amount of data is smaller. Therefore, this study found that determining the amount of disease data that can support model training requires attention to zero diagnosis days. Furthermore, the disease data set needs to satisfy the requirement that in the overall data, the number of days with zero confirmed cases is less than 40%. For example, 3 years of data consist of 1,095 days, so for any disease, if for at least 1,095 × 60% = 657 days, the number of confirmed cases is not 0, then the disease can be selected as a model study object. The fewer days with zero confirmed cases should be better to represent the disease as more common and easy to capture its epidemic trend. This means that when a disease is more common, its epidemic trend is easier to predict. Extreme cases are similar to cholera. During 2012–2020, only one patient was confirmed to have cholera, while the other months had zero cases. Thus, the model for cholera cannot be learned and predicted. Therefore, this study used tuberculosis, viral hepatitis, syphilis, scarlet fever, other infectious diarrhea, influenza, and HFMD. Various model characteristics will directly affect the rationality and accuracy of the prediction results. Therefore, effective features should be selected flexibly as per the characteristics of different infectious diseases. For example, HFMD is an infectious disease among children and students [31]. The main group activity of teenagers is to attend school, so the feature of whether they are on holiday is added to describe social gathering activities. The main mode of transmission of tuberculosis is person-to-person respiratory transmission, so the feature of whether January and February contain Chinese New Year needs to be included in the infectious disease trend prediction consideration [32]. During the Spring Festival, on account of the increased concentration of family gatherings, infection and transmission of tuberculosis potentially increase, and more patients tend to delay their treatment [33].

### 5.4. Limitations

The limitations of this study are mainly due to the small amount of data and the fact that the characteristics of the diseases were not considered in the modeling. On the one hand, this study focuses on early warning of trends in common and highly prevalent infectious diseases in hospitals. However, since some diseases such as plague, cholera, and Middle East respiratory syndrome are relatively rare (i.e., their annual incidence is less than 10 cases), their trends in individual hospitals are incidental and unpredictable. In the future, methods for predicting various types of infectious diseases can be developed based on real hospital medical record data in conjunction with the National Data Center for Public Health Sciences. Early warning and monitoring of emerging unknown infectious diseases are also worth exploring. On the other hand, it is important to note that the MSRD model in this study can calculate data characteristics such as the number of confirmed diagnoses, date, and temperature. However, the MSRD model cannot model the characteristics of the infectious disease itself or the characteristics of the infection, which still needs to be further explored.

It is also necessary to explain that the prediction of each day is based on the number of confirmed cases on the previous day and the meteorological forecast data for the next day. To predict results for Day *T* + 1, we need to use the actual number of confirmed cases on Day *T* and the climate data of Day *T* + 1. The mean value of historical temperature in the same period is used to replace the meteorological data of long-span continuous prediction. If we make a continuous prediction for the future, each prediction depends on the prediction results of the number of cases in the previous round. However, there are inevitable errors in each prediction. Thus, the continuous prediction will lead to error accumulation, and the longer the time span of continuous prediction, the larger the error accumulation and the lower the accuracy. In order to alleviate the issue of error accumulation, the historical average temperature of the same period is used in the prediction of a long-time span in the future because the actual temperature characteristics cannot be obtained, resulting in an a priori error. An alternative method with higher accuracy could be adopted to estimate the temperature. However, error accumulation occurs because the diagnosis case predicted in a certain time step needs to be used in the succeeding time step. As an alternative, the importance of the characteristics of the diagnosis number in the model construction could be reduced so that a minor fluctuation in the diagnosis number will not have a large impact on the prediction results.

## 6. Conclusions

In this study, we proposed an MSRD model to predict infectious disease trends in hospitals. Experimental results show that the proposed approach outperforms the SEIR model. The shortcomings of the SEIR model in hospital infection prediction were also elucidated. We also compare several neural network methods, such as DNN, LSTM, GRU, Bi-LSTM, and machine learning methods, such as SVM, Lasso regression, and Bayesian, and demonstrate that the MSRD method outperforms the above approaches. MSRD extracts the features of training data through a time window, avoiding the overfitting problem caused by long time series and the practical application of nonflexibility. In addition, the fitting ability of the model is improved by combining the output of each time step with the corresponding original input. The impact of common infectious diseases predicted by the model is consistent with the actual high prevalence of infectious diseases. The model combines hospital data with data from external data sources using a combination of medical record information, climate, and crowd gathering to provide information support for rapid response and decision-making and to assist hospitals in early warning and prediction of infectious diseases. The model can be extended to all types of hospitals for infectious disease surveillance, helping to advance infectious disease surveillance and prediction, promoting the standardization of infectious disease management, and contributing to dynamic early warning of infectious diseases.

## Figures and Tables

**Table 1:** Comparison of trend prediction results of infectious disease.

Comparison of trend prediction MAE results of infectious disease							
	Influenza	HFMD	Viral hepatitis	Tuberculosis	Syphilis	Scarlet fever	Infectious diarrhea
MSRD	1.6252	0.5650	7.6897	1.2754	0.4974	0.2198	0.9564
Bi-LSTM	1.8185	0.6018	8.6141	1.4270	0.5565	0.2459	1.0701
DNN	1.9585	0.6403	9.2669	1.5369	0.5894	0.2734	1.1525
LSTM	1.8859	0.6402	8.9231	1.4999	0.5771	0.2550	1.1098
GRU	1.8731	0.6075	8.8633	1.4698	0.5732	0.2533	1.1022
SVM	1.7034	0.8923	8.1596	1.3367	0.5213	0.2303	1.0024
Lasso	1.9342	0.8864	9.1515	1.5178	0.5920	0.2615	1.1382
Bayes	2.0652	0.6759	9.7713	1.6206	0.6320	0.2792	1.2152

Comparison of trend prediction RMSE results of infectious disease							
	Influenza	HFMD	Viral hepatitis	Tuberculosis	Syphilis	Scarlet fever	Infectious diarrhea

MSRD	3.8607	0.6069	9.1090	1.5705	1.7066	0.4459	1.253
Bi-LSTM	3.8588	0.8079	9.1744	1.5897	1.7177	0.4556	1.2663
DNN	3.8899	0.9209	9.3685	1.5924	1.7315	0.4492	1.2665
LSTM	3.8958	0.9170	9.2826	1.6048	1.7241	0.4499	1.2684
GRU	3.8763	0.8043	9.2361	1.5868	1.7155	0.4677	1.2620
SVM	3.8667	1.0531	9.4132	1.5829	1.8112	0.4565	1.2589
Lasso	4.5932	1.0859	10.9443	1.8903	2.0327	0.5305	1.4954
Bayes	3.9344	0.8462	9.3746	1.6106	1.7412	0.4544	1.2809

## Data Availability

The data that support the findings of this study are available from the corresponding author upon reasonable request.

## Abbreviations

MSRD: Multi self-regression deep
MAE: Mean absolute error
SEIR: Susceptible-exposed-infected-removed
NIDIMS: National infectious diseases information monitoring system
SEIRD: Susceptible-exposed-infected-recovered-die-hard-infected
ARIMA: Autoregressive integrated moving average
LSTM: Long short-term memory
CDC: National center for disease control and prevention
HFMD: Hand-foot-and-mouth disease
SVM: Support vector machine
DNN: Deep neural network
Bi-LSTM: Bi-directional long short-term memory
GRU: Gated recurrent unit.

## Supplementary Materials

Supplementary Materials Supplementary Material 1: Supplementary Figure 1: MSRD model predicts the epidemic situation of influenza in 2021; the green line is real data, and the red line is predicted data. Supplementary Figure 2: MSRD model predicts the epidemic situation of HFMD in 2021; the green line is real data, and the red line is predicted data. Supplementary Figure 3: MSRD model predicts the epidemic situation of viral hepatitis in 2021; the green line is real data, and the red line is predicted data. Supplementary Figure 4: MSRD model predicts the epidemic situation of tuberculosis in 2021; the green line is real data, and the red line is predicted data. Supplementary Figure 5: MSRD model predicts the epidemic situation of syphilis in 2021; the green line is real data, and the red line is predicted data. Supplementary Figure 6: MSRD model predicts the epidemic situation of scarlet fever in 2021; the green line is real data, and the red line is predicted data. Supplementary Material 2: Supplementary Figure 7: Process of getting the best parameters of MSRD.
