# Supplementary material for: Early Warning of Infectious Diseases in Hospitals Based on Multi-Self-Regression Deep Neural Network
Source: J Healthc Eng. 2022 Aug 18;2022:8990907. doi: 10.1155/2022/8990907 (PMC9410942; doi:10.1155/2022/8990907)
Supplement: Supplementary Materials — Supplementary Material 1: Supplementary Figure 1: MSRD model predicts the epidemic situation of influenza in 2021; the green line is real data, and the red line is predicted data. Supplementary Figure 2: MSRD model predicts the epidemic situation of HFMD in 2021; the green line is real data, and the red line is predicted data. Supplementary Figure 3: MSRD model predicts the epidemic situation of viral hepatitis in 2021; the green line is real data, and the red line is predicted data. Supplementary Figure 4: MSRD model predicts the epidemic situation of tuberculosis in 2021; the green line is real data, and the red line is predicted data. Supplementary Figure 5: MSRD model predicts the epidemic situation of syphilis in 2021; the green line is real data, and the red line is predicted data. Supplementary Figure 6: MSRD model predicts the epidemic situation of scarlet fever in 2021; the green line is real data, and the red line is predicted data. Supplementary Material 2: Supplementary Figure 7: Process of getting the best parameters of MSRD. [file 8990907.f1.pdf]

Supplementary material 1

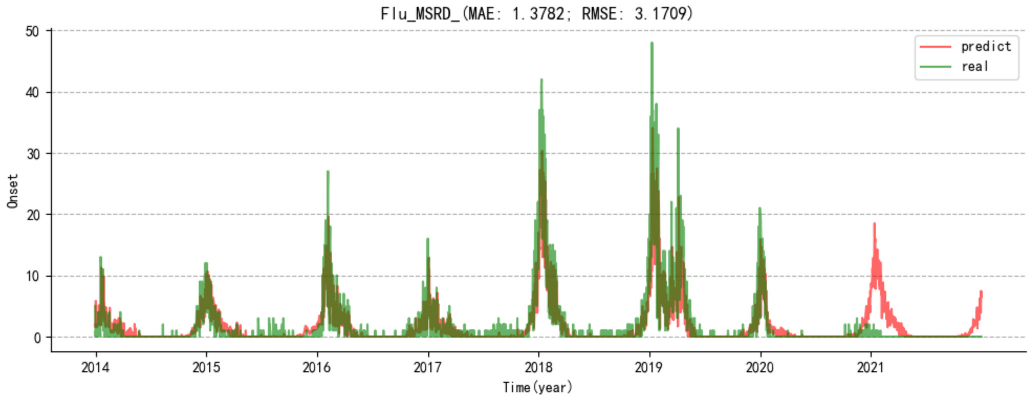

Supplementary figure 1. MSRD model predicts the epidemic situation of influenza in 2021; the green line is real data and the red line is predicted data

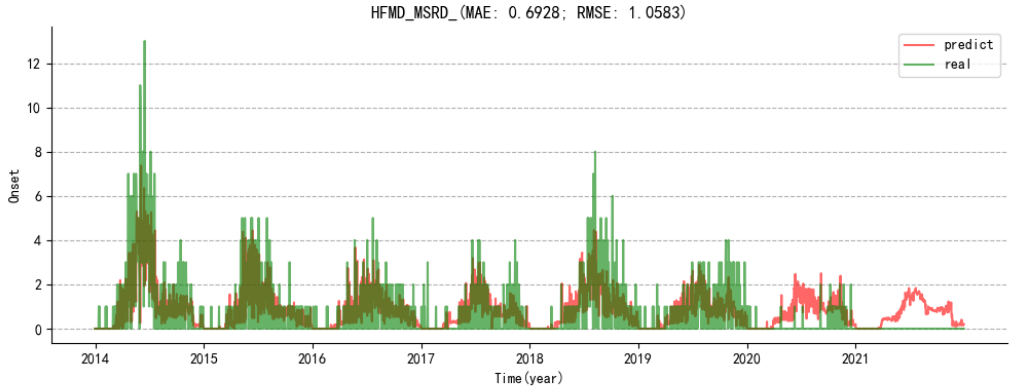

Supplementary figure 2. MSRD model predicts the epidemic situation of HFMD in 2021; the green line is real data and the red line is predicted data

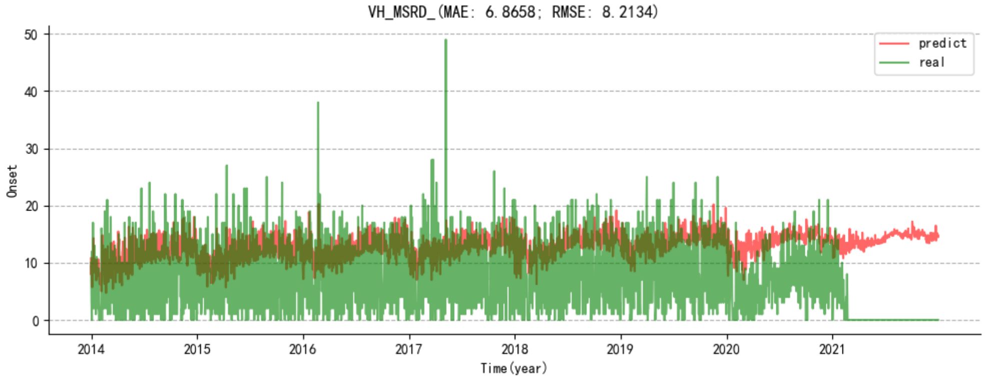

Supplementary figure 3. MSRD model predicts the epidemic situation of viral hepatitis in 2021; the green line is real data and the red line is predicted data

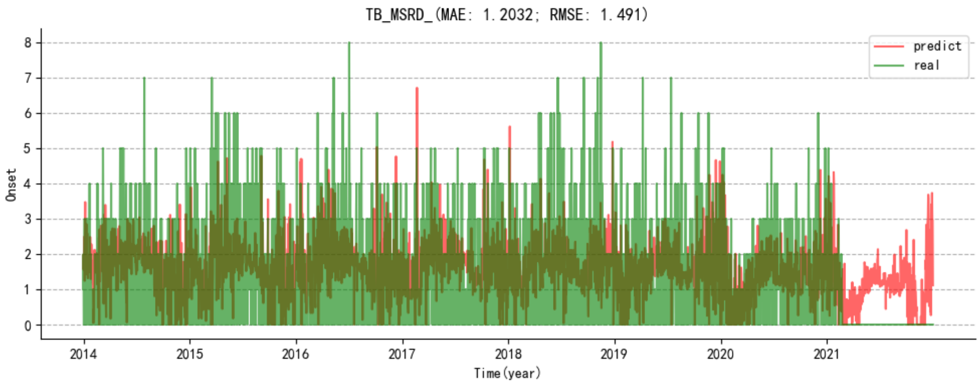

Supplementary figure 4 MSRD model predicts the epidemic situation of tuberculosis in 2021; the green line is real data and the red line is predicted data

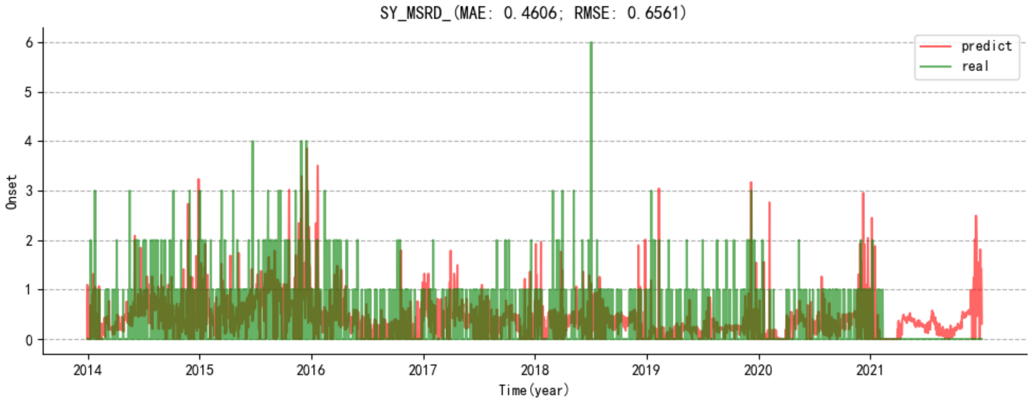

Supplementary figure 5. MSRD model predicts the epidemic situation of syphilis in 2021; the green line is real data and the red line is predicted data

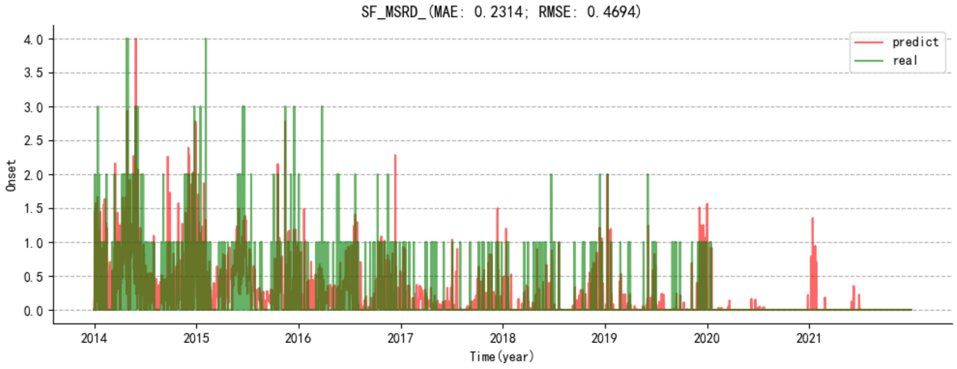

Supplementary figure 6. MSRD model predicts the epidemic situation of scarlet fever in 2021; the green line is real data and the red line is predicted data

Supplementary material 2

Sliding window width	Number of LSTM neurons	Number of feedforward neural networks	Learning rate		Sliding window width	Number of LSTM neurons	Number of feedforward neural networks	Learning rate		Sliding window width	Number of LSTM neurons	Number of feedforward neural networks	Learning rate		Sliding window width	Number of LSTM neurons	Number of feedforward neural networks	Learning rate
3	8	64	0.00001		5	8	64	0.00001		7	8	64	0.00001		9	8	64	0.00001
			0.0001					0.0001					0.0001					0.0001
			0.001					0.001					0.001					0.001
			0.01					0.01					0.01					0.01
		128	0.00001				128	0.00001				128	0.00001				128	0.00001
			0.0001					0.0001					0.0001					0.0001
			0.001					0.001					0.001					0.001
			0.01					0.01					0.01					0.01
		256	0.00001				256	0.00001				256	0.00001				256	0.00001
			0.0001					0.0001					0.0001					0.0001
			0.001					0.001					0.001					0.001
			0.01					0.01					0.01					0.01
	16	64	0.00001			16	64	0.00001			16	64	0.00001			16	64	0.00001
			0.0001					0.0001					0.0001					0.0001
			0.001					0.001					0.001					0.001
			0.01					0.01					0.01					0.01
		128	0.00001				128	0.00001				128	0.00001				128	0.00001
			0.0001					0.0001					0.0001					0.0001
			0.001					0.001					0.001					0.001
			0.01					0.01					0.01					0.01
		256	0.00001				256	0.00001				256	0.00001				256	0.00001
			0.0001					0.0001					0.0001					0.0001
			0.001					0.001					0.001					0.001
			0.01					0.01					0.01					0.01
	32	64	0.00001			32	64	0.00001			32	64	0.00001			32	64	0.00001
			0.0001					0.0001					0.0001					0.0001
			0.001					0.001					0.001					0.001
			0.01					0.01					0.01					0.01
		128	0.00001				128	0.00001				128	0.00001				128	0.00001
			0.0001					0.0001					0.0001					0.0001
			0.001					0.001					0.001					0.001
			0.01					0.01					0.01					0.01
		256	0.00001				256	0.00001				256	0.00001				256	0.00001
			0.0001					0.0001					0.0001					0.0001
			0.001					0.001					0.001					0.001
			0.01					0.01					0.01					0.01

Supplementary figure 7. Process of getting the best parameters of MSRD
